# Analysis of adult men’s knowledge in the area of male fertility in relation to selected lifestyle factors

**DOI:** 10.1038/s41598-026-45648-1

**Published:** 2026-03-25

**Authors:** Anna Liliana Dakowicz, Anna Justyna Milewska, Dominik Nowakowski, Adrianna Zańko, Michał Pawłowski, Justyna Skórka, Marcin Warpechowski, Magdalena Skowrońska, Robert Milewski

**Affiliations:** 1 Department of Biostatistics and Medical Informatics, Medical University of Bialystok, Bialystok, 15-295 Poland; 2 Doctoral School, Medical University of Bialystok, Bialystok, 15-276 Poland

**Keywords:** Diseases, Health care, Medical research, Risk factors

## Abstract

The aim of this study was to assess the level of knowledge among adult men regarding male fertility, with particular emphasis on selected factors connected with medical and lifestyle-related aspects of fertility. The results provide a strong basis for further research aimed at identifying knowledge gaps in male infertility and developing effective educational strategies. The study was performed in 156 men of reproductive age. An anonymous questionnaire developed by the authors was used, consisting of two sections: a demographic section and 25 single-choice questions assessing the subjects’ knowledge in the area of medical and lifestyle-related aspects of male fertility. The results revealed the existence of a weak positive correlation between the level of knowledge about male fertility and age. Moreover, the level of fertility-related knowledge was higher in those participants who had an education in a field connected with medicine as well as in men suspected of or treated for infertility. Another novel finding is that although many respondents expressed an interest in health—as evidenced in their declaring the use of dietary supplements—this was not reflected in their performance in the questionnaire. This highlights the gap between health-related decisions and limited fertility literacy. In general, the findings show that the participants’ level of knowledge concerning male fertility was inadequate in most areas—both medical and lifestyle-related—covered by the questionnaire. Based on the findings of the study, relevant recommendations for educational initiatives were proposed, which could potentially improve fertility awareness among men.

## Introduction

According to the World Health Organization (WHO), infertility is defined as a condition characterized by the inability to conceive for at least one year despite regular unprotected sexual intercourse without the use of contraception. Infertility affects approx. 10–18% couples worldwide, which makes it an issue of global importance. In Europe, the proportion of population who have experienced infertility is approximately 16.5%^1^. In Poland, approximately 10–15% couples face infertility, which represents over a million couples in total. These numbers are expected to gradually increase in the coming years, partly due to the increasing age at which people decide to plan having children^2,3^. Data obtained from fertility clinics show that infertility treatment is most often sought by women. However, as infertility involves both partners, it is crucial to perform diagnostics in both the female and the male partner, especially given that male factors are estimated to be the cause in approximately half of all infertility cases^4^. Interestingly, studies suggest that male fertility is an indicator of a man’s overall health, with men diagnosed with fertility problems being more likely to suffer from other health issues, such as cardiovascular diseases, diabetes, testicular cancer, or prostate cancer^5,6^.

Growing evidence suggests that low awareness of male fertility may contribute to delays in the diagnosis and treatment of infertility^7^. Men often do not perceive their own reproductive health as an important factor in family planning, which can lead to delayed andrological evaluation and delayed recognition of modifiable risk factors related to lifestyle. Consequently, male fertility assessment is often postponed until treatment attempts focused solely on the female partner are unsuccessful, which can prolong the time to a correct diagnosis and implementation of appropriate management^8^.

Men often find it more difficult to accept and cope with the realization that they may have fertility problems^9^. They may perceive it as a personal failure and are thus reluctant to discuss it. For many men, the ability to have children holds significant personal value, and the prospect of infertility can lead to considerable disappointment. In addition, feelings of shame are sometimes experienced by men, as they mistakenly believe themselves to be isolated. This may in turn lead to a lowered sense of self-esteem, apathy, or withdrawal. Indeed, observations from infertility treatment clinics indicate that men facing infertility often require increased emotional support from their close ones or specialists. This situation is also exacerbated by the fact that the public perception of the issue primarily focuses on female infertility, while male infertility tends to be overlooked^9^. This state of affairs has only started to improve recently, owing to growing public awareness and various social campaigns around the world aimed at educating men about fertility such as “Fertile Polish Man”. The campaign revealed that as many as 94% of those surveyed had never undergone semen analysis, even though their daily habits negatively affected semen quality^10^. Such results corroborate the fact that the topic of male fertility needs greater attention than it currently receives.

Although infertility is a challenging condition, it is in many cases treatable. The diagnostic process focuses on couples struggling with infertility and involves both partners. In men, it starts with the assessment of semen quality after 2–7 days of sexual abstinence. The ejaculate is evaluated for volume, sperm count, and motility, among other parameters^4,11,12^. Semen analysis is a key element in the diagnosis of male infertility, constituting the basis for further diagnostic and therapeutic proceedings^4^. In recent years, a notable decline in the number of sperm with normal morphology and motility has been observed, while the number of sperm unable to fertilize an egg has increased. These trends should be considered as a meaningful problem in context of fertility, since lowered sperm parameters translate into reduced chances of successful pregnancy outcomes. The contributing factors include unhealthy lifestyle choices—including diet—prolonged stress, overweight and obesity, testicular diseases, systemic diseases, or other environmental factors such as wearing tight underwear^13^. Other potential causes include being over 40 years of age, mechanical testicular damage, or complications following inflammatory diseases in the reproductive organs. In some cases, the causes of infertility remain undetermined due to limited access to certain diagnostic tests. It is also important to note that many conditions negatively affecting male fertility may not present visible symptoms, which is why consultation with a specialist is crucial^11,12,14,15^.

Although various methods to improve semen parameters are proposed, causal relationships between a particular intervention and its desired outcome in the form of improved semen quality are still unclear. One of the areas that shows promising results are lifestyle modifications, with research showing that they may play a considerable role as far as improving male fertility is concerned. Commonly recommended interventions include the cessation of alcohol consumption, smoking, and other harmful substances. Maintaining an appropriate body weight and following a balanced diet—high in protein, low in fat, and rich in fruits, vegetables, and whole grains—that meets the patient’s energy requirements is also essential^16^. Other lifestyle changes may include regular performing physical activity, stress management, and sufficient, high-quality sleep. It must be emphasized that while the impact of the aforementioned modifications on the overall well-being is a well-studied area, their influence on semen parameters is under-researched and well-designed prospective studies are needed to establish associations^14,17^.

As far as the relationship between dietary modifications and male fertility is concerned, the adequate intake of vitamins and minerals plays a crucial role. It is recommended that meals rich in zinc and selenium should be consumed. These minerals significantly affect proper sperm maturation, with their deficiencies leading to a decrease in testosterone levels and erectile dysfunction. Vitamin C intake is also important, as it has been shown to be associated with extended sperm lifespan, increased motility, and protection of sperm DNA from damage caused by oxidative stress^18^. In addition, supplementation with inositol should be considered, as it contributes to improved semen quality by regulating its composition and aids sperm movement to the epididymis. Combined with antioxidants such as vitamin C, inositol has been sown to positively affect sperm motility, concentration, and the number of sperm with normal morphology^19^. Moreover, folate, vitamin D, vitamin B6, and vitamin B12 are also essential for male fertility, being responsible for the proper structure of sperm DNA and adequate motility. Deficiencies in these vitamins can also lead to a reduction in sperm count in semen^20,21^. In general, lifestyle modifications constitute an effective method for improving male fertility, although these changes must be introduced in a thoughtful and targeted manner.

Although many lifestyle factors that influence fertility are modifiable, self-awareness among men is crucial in order to make it possible to introduce effective interventions. As studies on couples’ knowledge of infertility clearly indicate that women tend to be both more knowledgeable and involved in the subject, educating men in the area is increasingly often seen as necessary for the improvement of fertility treatment outcomes. Knowledge of the causes of infertility and its preventive and remedial measures could significantly reduce the scale of the problem. Hence, awareness from both partners is a crucial step in the infertility treatment process, but also in maintaining optimal fertility^22^.

Although there are indications that men’s knowledge about fertility issues may be limited, existing studies do not provide a clear consensus on the extent or nature of this knowledge gap. For this reason, and due to the increasing active involvement of the male factor in the contexts of maintaining fertility or its treatment, the aim of this study was to assess the level of knowledge of adult men about male fertility in relation to selected elements connected with lifestyle and health-related factors.

## Materials and methods

### Questionnaire and participants

This questionnaire-based study was performed using an anonymous online form completed by the participants. The questionnaires were addressed to adult men of diverse demographic and professional backgrounds. A total of 156 men completed the survey. No personal data of the participants were collected nor processed. According to applicable Polish law, Bioethics Committee approval for this study was not required.

The survey instrument was developed based on a review of the literature related to infertility and lifestyle. The anonymous questionnaire was designed by a team consisting of, among others, specialists in dietetics and infertility. During the questionnaire development phase, a pilot survey was conducted to verify the understanding, clarity, and intuitiveness of the questions. The submitted comments and suggestions were incorporated into the final version.

At the beginning of the questionnaire, a disclaimer was placed that detailed the necessary information regarding the purpose of the study as well as assurances of the anonymity of responses and voluntary participation in the study.

The items included in the questionnaire focused on two aspects. One of them was assessing lifestyle-related elements selected on the basis of the latest research findings concerning the impact of lifestyle, including nutrition, on male fertility. The other aspect focused on the respondents’ general knowledge concerning the medical aspects of infertility.

The first part of the questionnaire (Part I) included items focused on demographic data such as age, level of education, occupation, relationships, and children. It also included a question whether the respondent’s education or profession was related to medicine, and questions ascertaining whether the respondent was suspected of or treated for infertility.

The second part of the questionnaire (Part II) contained 25 single-choice questions concerning the respondents’ knowledge related to infertility, especially male infertility. This part included 10 medical (no. 1, 3, 7, 10, 12, 14, 15, and 18–25) and 15 lifestyle-related (nutrition, physical activity, supplementation, etc.; no. 2, 4–6, 8, 9, 11, 13, 16, and 17) items. Each correct answer was awarded 1 point, with 0 points assigned for an incorrect answer. It was possible to obtain a total of 25 points in the questionnaire.

### Statistical analysis

The normality of distribution of continuous variables was tested using the Shapiro-Wilk test. Due to the lack of normality of distribution of the analyzed variables, nonparametric tests were used. For comparisons between two independent groups the U Mann–Whitney test was used. The asymptotic significance test of Spearman’s correlation coefficient was used for correlations between continuous variables. The reliability of the knowledge test was assessed using the Kuder–Richardson Formula 20 (KR-20), which is appropriate for instruments composed of dichotomous items (correct/incorrect responses). The test consisted of 25 items addressing a single thematic domain, and the obtained KR-20 coefficient was 0.78, indicating good internal consistency of the instrument. Statistical significance was set at *p* < 0.05. The results were analyzed using Statistica 13.3 (TIBCO Software, Palo Alto, CA, USA) and R 4.4.2 (R Core Team, R Foundation for Statistical Computing, Vienna, Austria).

## Results

A total of 156 men aged 18–74 completed the survey, 90% of whom were aged 30 or younger. At the time of participation, 51% were in a relationship while 49% were single (47% bachelors and 2% after a long-term relationship). Only 8% of the respondents had children. Most respondents reported secondary (60%) or higher education (33%) and lived in cities with populations above 50,000 inhabitants (72%). Additionally, 14% had a medical background and 3% reported suspected or diagnosed fertility problems. The characteristics of the study group are presented in Table 1.

**Table 1:** Demographic characteristics of the group.

Demographic feature	Category	*n*	%
Relationship status	Bachelor	74	47%
	In a relationship	79	51%
	After a long-term relationship	3	2%
Character of the work	Manual labor	30	19%
	Moderately active job	36	23%
	Sedentary job	54	35%
	Unemployed	36	23%
Education	Primary	5	3%
	Secondary	93	60%
	Vocational	6	4%
	Higher	52	33%
Residence	Village	24	15%
	City up to 50,000 inhabitants	20	13%
	City with over 50,000 inhabitants	112	72%
Having children	No	143	92%
	Yes	13	8%
Medical profession or education	No	134	86%
	Yes	22	14%
Suspected or diagnosed fertility problems	No	151	97%
	Yes	5	3%
Physical activity	At all	4	3%
	Occasionally	34	22%
	Several times a month	22	14%
	Once a week	14	9%
	Several times a week	72	46%
	Every day	10	6%

**Fig. 1:**
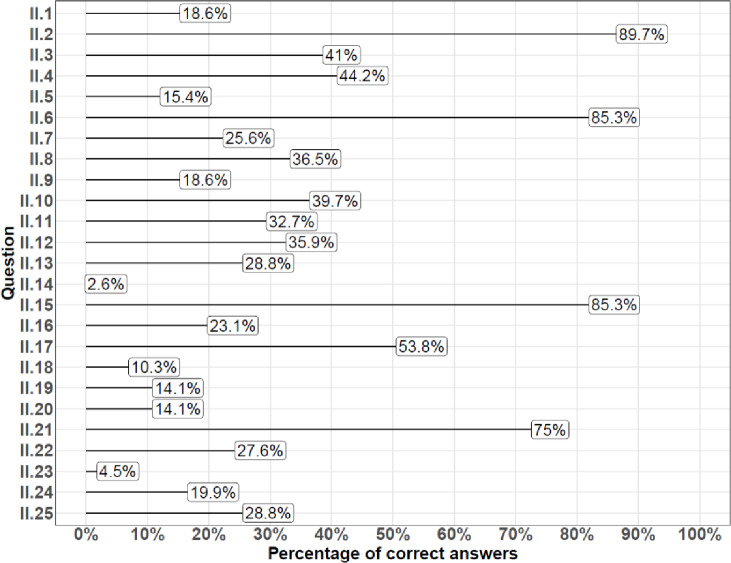
Percentages of correct answers scored for each of the questions included in the questionnaire. Each line corresponds to a single test item; the percentage values indicate the proportion of respondents who provided a correct answer. The variability in the proportion of correct responses reflects the differences in item difficulty.

Figure 1 shows the percentages of correct answers for each of the questions in Part II the questionnaire. Only 5 out of 25 questions (20%) were answered correctly by the majority of the respondents. The four questions with the greatest numbers of positive answers (2, 6, 15, and 21) were evenly divided between lifestyle-related and medical topics. The two questions with the lowest numbers of positive answers (14 and 23) were both medical questions.

The following items had the highest percentages of correct answers:

II.2. Can eating habits affect fertility? (89.7%, lifestyle-related question)

II.6. Can too little physical activity negatively impact male fertility? (85.3%, lifestyle-related question)

II.15. How many sperm are needed to fertilize an egg cell? (85.3%, medical question)

II.21. What are the structures called through which sperm are transported from the testes? (75%, medical question)

The following items had the highest percentages of incorrect answers:

II.14. What is the minimum percentage of sperm that must have normal morphology for this parameter to be considered normal? (2.6%, medical question)

II.23. Which of the following structures is essential for semen production? (4.5%, medical question)

**Fig. 2:**
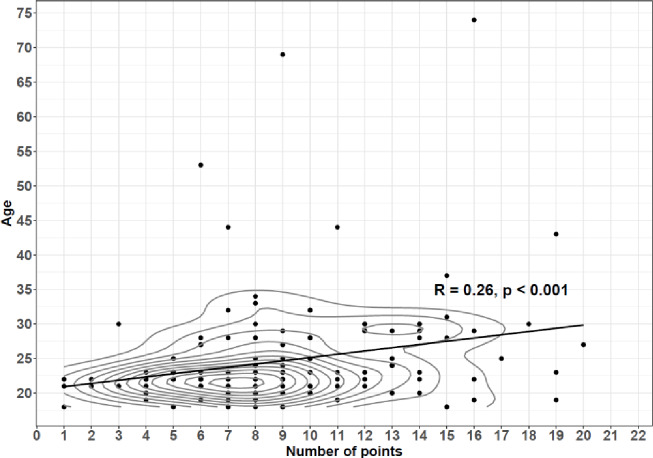
Correlation between the number of points obtained in the test and the age of the respondents. The black dots represent individual observations. The solid line depicts the best-fit linear regression line, illustrating the direction and nature of the association between the analyzed variables. The gray contour lines indicate areas of the highest concentration of observations.

A weak (*R* = 0.26), positive correlation (*p* < 0.001) was found between the number of points scored by the respondents and their age. The results are presented in Fig. 2.

**Fig. 3:**
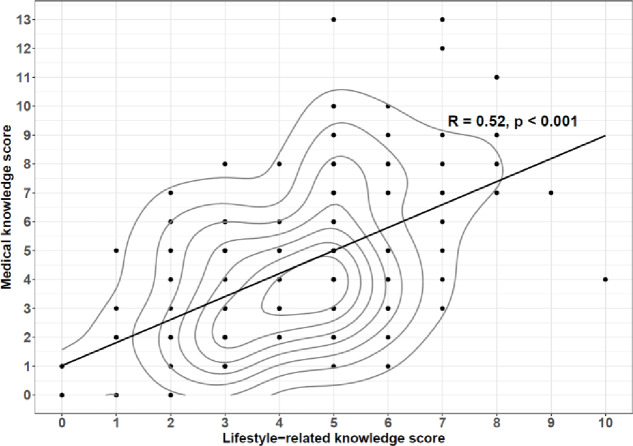
Correlation between the numbers of points scored by the respondents in lifestyle-related and medical questions. The black dots represent individual observations. The solid line indicates the best-fit linear regression line, illustrating the direction and strength of the association between the two knowledge domains. The gray contour lines depict areas of higher concentration of observations.

A strong (*R* = 0.52), positive correlation (*p* < 0.001) was found between the number of points scored in lifestyle-related questions and the number of points scored in medical questions, indicating that the participants did not tend to exhibit a higher level of knowledge in either of the tested areas. The results are presented in Fig. 3.

**Fig. 4:**
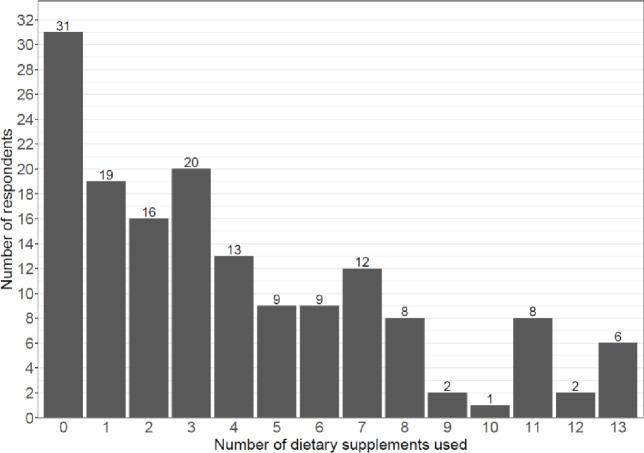
The prevalence of supplementation among the respondents. The horizontal axis represents the number of dietary supplements used by each respondent (calculated based on multiple-response answers), while the vertical axis represents the number of respondents.

The histogram presented in Fig. 4 illustrates the prevalence of supplementation among the respondents. A large majority (80.1%) of the respondents use supplements, with most of them (84.8%) opting for multi-supplements, i.e., those containing more than two ingredients.

No statistically significant associations were found between characteristics of the study group such as age, education/profession, and whether they were suspected of or treated of fertility and those characteristics of the supplemented ingredients that are known to be associated with fertility such as their oxidative potential.

**Fig. 5:**
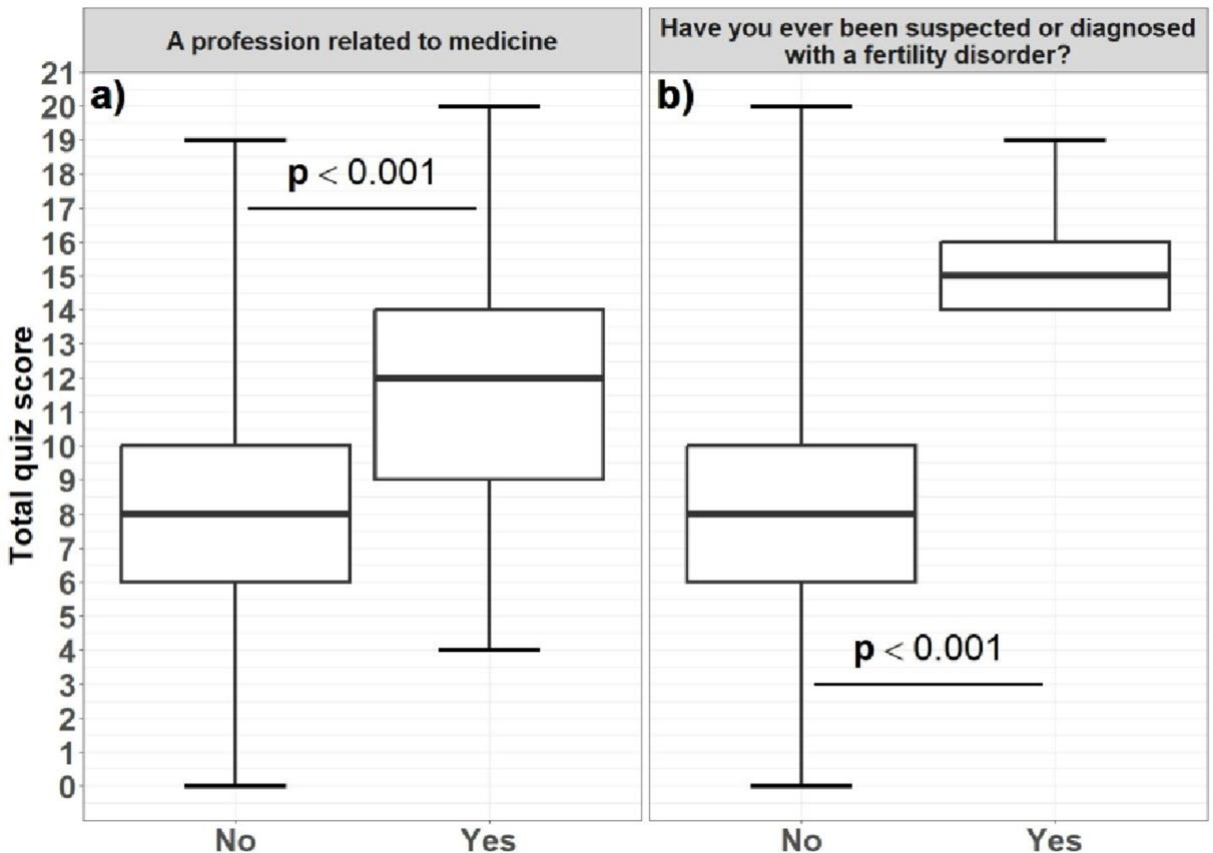
Distribution of points scored on the questionnaire between participants whose education or profession was related to medicine and those whose education or profession was not related to medicine **(a**); and between participants suspected of or treated for infertility and those not suspected of infertility issues **(b)**.

Figure 5 illustrates the distribution of points scored on the questionnaire depending on whether the respondent’s education or profession was related to medicine or not and whether the respondent was suspected of or treated for infertility. For each analysis, separate boxplots are presented to illustrate the distribution of the total test scores in the compared subgroups. The horizontal line within each box represents the median, the lower and upper edges of the box correspond to the first and third quartiles (Q1–Q3), and the whiskers indicate the range of observed values (minimum – maximum). A significant difference (*p* < 0.001) in the level of knowledge was found between people connected with a medical profession and those whose education nor profession was not related to medicine (a) and between men suspected of or treated for infertility and those who were not suspected of infertility issues (b). Men not related to medical professions and those who were not suspected of infertility issues achieved lower results.

## Discussion

The aim of this study was to assess the level of knowledge of adult men about male fertility. The results of the study indicate an overall low level of knowledge in the studied area, with older respondents scoring slightly better results on the administered questionnaire. In addition, significant differences in the level of knowledge were found between men whose education or profession was connected with medicine and those whose education or profession was unrelated to medicine, as well as between men suspected of or treated for infertility and those without such a suspicion or diagnosis. As far as the tested categories of questions are concerned, the respondents tended to answer correctly in similar proportions regardless of whether a particular question was medicine- or lifestyle-related. Interestingly, the relatively low level of the respondents’ knowledge about male fertility did not correspond with the high propensity for using supplementation indicated in the questionnaires. As this type of behavior may be interpreted as suggesting a certain level of awareness of the need to introduce lifestyle-related interventions conducive to overall health improvement, it seems at odds with the low level of medical knowledge reflected in the results of the study. This low awareness among men may result in shifting the infertility responsibility toward the female partner.

The relatively low level of knowledge about their fertility among men is a global phenomenon, reflected across various populations and regions—independent from cultural, social, and macroeconomic factors^23,24^. In this respect, the results of this research are consistent with findings of other studies conducted in the last two decades, indicating that an average male is equipped neither with extensive knowledge about male reproductive health nor about the factors that influence fertility^24^. In a study performed in childless men by Daniluk & Koert^25^, at least half of the participants correctly answered only 4 out of 20 questions regarding male reproductive health. Although the results of that study and the present research cannot be directly compared due to differences in the sets of questions and potentially varying levels of difficulty, the findings are similar: in the present study, only 20% of answers across the whole tested group were correct. Similar results have been reported by Quach & Librach^26^, who showed that younger persons—including secondary school students—are characterized by a low level of knowledge about fertility, with 22.9% of the participants being unable to correctly identify the definition of infertility. Interestingly, as much as 5% (39 persons) of the respondents did not know the meaning of the word ‘infertility’ and answered “I don’t know” to all the questions or gave no answer. Not only does this situation signifies knowledge gaps, but it also reflects the indifferent attitude of young adults towards the topic of infertility. Both the results of this study and the literature data clearly indicate that knowledge deficits regarding male fertility are systemic in nature and occur regardless of the cultural, social, or life-stage context.

The results also show that a man’s age is one of the factors associated with a higher level of knowledge about male fertility. Although the overall knowledge about male fertility is low across the whole age spectrum, the literature data report increased awareness of factors that influence fertility among older persons^24,27^. Likewise, the previously discussed study performed in secondary school students by Quach & Librach^26^ confirms that the knowledge and attitudes concerning infertility in this group are inadequate. This is further corroborated by qualitative research findings about male fertility-related issues increases with age—on the basis in-depth interviews with young adult men about their views on the decline in fertility with age, Law^28^ makes a general conclusion that older men have greater knowledge about male fertility compared to younger males. It can thus be stated that as older men more often face challenges related to their own fertility, this encourages them to seek information and most probably contributes to their increased level of knowledge.

Another factor that has an impact on men’s knowledge about male fertility is whether they have connections with medicine—through education or professional work—or not. Greater awareness of reproductive issues among health care professionals is not a surprising finding, supported in the literature data. For instance, in an American study involving nearly 2,700 students from three universities, in particular those enrolled at courses connected with medicine and related fields, male respondents achieved an average of 61–63% correct answers to questions about factors affecting fertility^29^. Even accounting for the differences in the difficulty level of test questions between studies, this is a much greater score compared to those reported for the general population of men, e.g., by Daniluk & Koert^25^. Nouri et al.^30^ confirm that students enrolled at courses connected with health care possess better knowledge about fertility. However, some studies report that the knowledge of health care professionals about fertility may in fact be inadequate^31^ or not differ substantially from the general population^32^. Such inconsistencies may be explained by differences in the types of persons connected with medicine participating in the various studies as not all professions in the medical field require having knowledge about fertility. In this respect, the available literature data are consistent with the results obtained in this study, i.e., although the knowledge about male infertility among men with a background in medicine is overall greater compared to persons unrelated to medical fields, it may still be seen as inadequate and requiring educational interventions. On the other hand, the results of the present study indicate that—possibly contrary to expectations—persons connected with medicine should not be the primary target groups of educational efforts aimed at increasing fertility knowledge.

A higher level of knowledge about male fertility has also been observed among men suspected of or treated for infertility. Barron et al.^29^ indicated that men who reported having acquired formal or informal knowledge about health through prior exposure to the topic of infertility more often achieved higher scores on the knowledge test used in their study, which focused on factors affecting male fertility. The literature data also indicate that compared to fertile men, those who have contact with the issue of infertility have better awareness not only of general fertility-related topics but also of certain specific areas connected with fertility. Comparing the level of knowledge about male infertility and the acceptance of assisted reproductive technologies (ART) among fertile and infertile men in Senegal, Gaye et al.^33^ demonstrated that infertile men had a higher level of knowledge and greater acceptance of ART procedures compared to fertile men. Over half of the men diagnosed with infertility had a level of knowledge classified as ‘good’; infertile men also showed greater motivation and engagement in expanding their knowledge compared to fertile men. According to Satir and Kavlak^34^, 68% of men affected by infertility searched for information about the treatment of their condition online, with approximately half of them then sharing the insights they had gained with their doctors during appointments. This indicates that direct contact with the trained staff of infertility clinics may be seen by men as a way to consolidate their knowledge and clarify doubts, suggesting that exposure to the issue of infertility is conducive to a higher level of knowledge about male fertility. For this reason——men who are suspected of or treated for infertility should not be the main focus of educational efforts in the area of fertility. Not only do they show greater knowledge than men who have no known fertility issues, but their contact with fertility clinics is already a credible source of information.

A further finding of the present study indicates that the respondents’ knowledge about infertility is relatively low in medical as well as lifestyle-related aspects of male fertility. While the greater relative difficulty of medical questions was an expected finding, the gaps in knowledge on lifestyle-related topics represent a less obvious result, indicating low awareness among respondents regarding the links between lifestyle choices and male fertility. The low level of men’s knowledge about the medical aspects of fertility is, unsurprisingly, well documented in the literature, which also provides examples of numerous misconceptions and knowledge gaps prevalent among men. For example, men often mistakenly equate their potency with fertility, assuming that if sexual functions such as erection and libido are normal, then the person experiences no problems with fertility^23,24^. It is also worth noting that men have been shown not to regard using anabolic steroids—which are a common element of a lifestyle that is focused on physical fitness—as a potential cause of issues with fertility, which is inconsistent with the fact that these substances can impair sperm production^35^. The aforementioned examples confirm the presence of deeply rooted misconceptions among men regarding both the medical and lifestyle factors influencing male fertility.

The gaps in knowledge regarding the various issues related to male infertility should also be considered in relation to men’s tendency to use supplementation, as demonstrated in the findings of the present study. The results suggest that despite the fact that men’s awareness of the impact of supplementation on reproductive health may actually be very limited, using supplements is indeed a common behavior. Despite the lack of substantive medical knowledge, over 80% of the respondents use supplementation, mostly in the form of multi-ingredient preparations. Not only does this raise concerns—especially when supplementation is not consulted with a doctor, dietitian, or pharmacist—but it also indicates that although men have a general awareness of the need for engaging in health-promoting behaviors, they apparently act without the knowledge necessary to make potentially effective targeted decisions. In other words, while spontaneous health-conscious actions are a positive trait, a systematic approach would be necessary for men to achieve the desired effect. This tendency is confirmed in the literature data, which show that although men take supplements, this is not always associated with a comprehensive lifestyle change or deeper knowledge^23,24^. In this context, although supplementation is a common practice among men, the rationale behind its use reveals a gap between intention and a focused, structured approach to procreative health.

It should be noted that the abuse of OTC medications and dietary supplements is a significant public health problem^36,37^. Poland is one of the countries with the most liberal regulations on OTC sales in the European Union, which results in a very wide and easy availability of non-pharmacy preparations and their intensive promotion in the media^38^, with as much as 70.6% of the country’s population declaring the use of dietary supplements^39^. This may suggest that the use of health-promoting products is often based on a socially entrenched belief that they are harmless, rather than on actual medical recommendations. As a consequence of this high level of medicalization, a belief may develop that resolving health problems—including those related to fertility—is possible without a proper medical diagnosis. In the reality of strong exposure to OCT drugs and dietary supplements, low fertility literacy does not facilitate a critical assessment of the validity of taking pharmaceuticals, further intensifying the phenomenon of their excessive use, especially in Poland. Hence, building awareness in this area through educational initiatives could correct misconceptions, limit the phenomenon of supplementation as self-medication^40^, and lead to well-informed decisions in the area of fertility.

Most importantly, the results of this study indicate a strong need for education regarding men’s knowledge about their fertility, including making rational lifestyle choices. The effectiveness of education is also evidenced in the higher test scores among men whose education or profession was connected with medicine as well as among men suspected of or treated for infertility, whose source of knowledge were fertility clinic staff members. Hence, as mentioned above, the results indicate that the two aforementioned groups should not be the primary focus of educational efforts. The effectiveness of education is demonstrated in the results of a recent literature review performed by Hammarberg et al.^24^ whose findings showed that all the analyzed educational interventions targeting men had led to increased knowledge, especially among younger individuals and those with lower education levels. According to Barron et al.^29^, knowledge about reproductive health should be widely promoted in various settings, including secondary schools, targeting not only women as the traditional primary audience, but also men. The level of knowledge about fertility is reflected in the couple’s procreative behavior, with Hoffman et al.^41^ emphasizing that lower knowledge is associated with a longer period of untreated infertility. This means that when facing difficulties in conception, better-educated couples seek specialist help more readily. Thus, a low level of knowledge may contribute to a passive attitude toward one’s reproductive health, including delays in diagnostic and therapeutic decisions^23,42^. Clearly, educational interventions translate into increased knowledge, which positively impacts procreative decisions, and thus fertility education should be catered not only to the age, but also to the differing professional backgrounds of the recipients.

At the same time, a problem connected with the lack of a structured model for the dissemination of knowledge about fertility should be emphasized. Over half of men declare that their preferred sources of information about fertility are doctors or socially responsible online resources^23,24^, which are obviously sources existing outside any formal educational structure. As widespread educational programs are uncommon, this means that the average man—one outside the medical community or contexts connected with infertility counseling and treatment—usually does not acquire even basic knowledge about reproduction. International recommendations advise building fertility literacy competencies from adolescence to adulthood, which may contribute to making informed lifestyle-related decisions that could reduce the incidence of infertility requiring treatment. As mentioned earlier, better-informed men more often introduce positive changes regarding lifestyle choices—modifying their diet, reducing substance use, or taking various preventive measures^4,24,43^. Crucially, it is recommended that messages concerning reproductive health should be simple, culturally understandable, and repeatedly reinforced^44^, whereas one-off campaigns may prove insufficient^45^. Education in the context of reproductive health should direct the awareness of teenagers and young adults, systematically reminding them about both common and less obvious deficits in men’s lifestyle. In this regard, a solution to the problem of low knowledge among men regarding fertility could be the implementation of formal, medically oriented education programs delivered through multiple channels, e.g., through social media or spread in places frequented by men of reproductive age, based on scientific evidence supported by authority, and with particular emphasis on effective lifestyle modifications. The findings of this study may also be useful for primary care physicians and gynecologists, as they emphasize the importance of early involvement of the male partner in the evaluation of infertility and consideration of a referral to an andrologist. Most importantly, such programs must focus on the general population, rather than men facing fertility issues or those connected with medical professions.

It is also worth mentioning that an important methodological challenge in assessing knowledge about fertility is the absence of standardized and validated knowledge tests. The use of non-standardized levels of difficulty and ill-defined ranges of questions make it difficult to compare results collected from different regions, countries, and continents, which may in turn lead to inconsistent or even contradictory conclusions. The literature data indicate the existence of a clear tendency to adapt questionnaires developed by other researchers, often without formal psychometric evaluation and with a high thematic variability of questions^46–48^. It also appears that certain discrepancies and inconsistencies in the results may stem from the variability of measurement tools, as opposed to actual existing differences. This issue could serve as a starting point for a discussion on the need to use validated instruments^47,49,50^.

The results of the study indicate an overall low level of men’s knowledge about their reproductive health, depending on age, but particularly on the following two factors: whether the man’s education or profession was related to medicine or not, and whether they were suspected of or treated for infertility. This may suggest that fertility education is still not sufficiently widespread. Most importantly, the overall state of knowledge about fertility among men remains relatively low, even among the aforementioned groups. These findings confirm the need to intensify educational efforts, which should be tailored to different social groups and implemented at multiple levels. Increasing men’s awareness of their role in the reproductive process, as well as education in reproductive health, may significantly contribute to the early diagnosis of infertility and the improvement of reproductive health.

## Conclusion

The results of this study demonstrate that the level of men’s knowledge concerning male fertility is generally low, both in medical and lifestyle-related aspects of fertility. However, certain groups of men were characterized by significantly greater knowledge gaps in this area than others. Inadequate knowledge concerning fertility was found to be associated with the age of respondents, as older participants scored slightly higher on the test, which may be attributed to their greater experience and earlier exposure to fertility-related topics. Furthermore, significant differences in the knowledge concerning male fertility were observed between individuals working in medical professions and those whose occupations were unrelated to medicine, with the former displaying a greater level of expertise. This may suggest that, as expected, working in a medical field provides a higher level of knowledge regarding male fertility. A similar result was obtained for man suspected of or treated for infertility, i.e., their level of knowledge concerning fertility was higher than that of men with no suspected fertility issues. It is also worth noting that although most respondents use supplements, which indicates an interest in health, their choices of supplements appear to be based more on a belief in their effectiveness rather than on reliable medical knowledge. Overall, the findings of the research may indicate a lack of understanding regarding reproductive health prevalent among men. Although it may be argued that educational efforts should focus primarily on medical professionals—as those responsible for the treatment of infertility—or on individuals directly affected by the problem—as the recipients of care. However, the findings of this study indicate that both these groups already demonstrate a higher level of knowledge, which suggests that educational initiatives should target the broader population, rather than focusing exclusively on healthcare providers or those directly impacted by the infertility. Such educational initiatives could include the promotion of informed choices regarding lifestyle, supplementation, and infertility treatment. They could help raise men’s knowledge about fertility, make it possible for them to introduce well-informed interventions aimed at improving fertility, and consequently have a positive impact on the population’s overall reproductive health.

### Limitations of the study

Due to the use of an online questionnaire completed anonymously and independently by participants, it was not possible to entirely rule out errors resulting from misunderstandings or misinterpretation of the questions, including recall or hypothetical bias. Additionally, the voluntary nature of participation may have contributed to an overrepresentation of individuals with a particular interest in health and lifestyle-related topics. Despite these limitations, online recruitment made it possible to reach a geographically more diverse group of respondents that the alternatives and is an approach commonly used in exploratory research on health knowledge and attitudes. Another limitation is the use of a self-developed questionnaire. The lack of standardization may have impacted the validity and reliability of responses, especially when compared with other studies. Furthermore, due to the sensitive nature of the topic, some respondents may have chosen not to provide fully honest responses or may have skipped questions they deemed too personal.

## Data Availability

The datasets generated during and/or analysed during the current study are available from the corresponding author on reasonable request.
